# Extending life for people with a terminal illness: a moral right and an expensive death? Exploring societal perspectives

**DOI:** 10.1186/s12910-015-0008-x

**Published:** 2015-03-07

**Authors:** Neil McHugh, Rachel M Baker, Helen Mason, Laura Williamson, Job van Exel, Rohan Deogaonkar, Marissa Collins, Cam Donaldson

**Affiliations:** Yunus Centre for Social Business and Health, Glasgow Caledonian University, Glasgow, Scotland UK; Institute for Applied Health Research, Glasgow Caledonian University, Glasgow, Scotland UK; Institute of Health Policy & Management, Erasmus University, Rotterdam, Netherlands

**Keywords:** Social values, Life extension, Terminal illness, Societal perspectives, Health policy, Ethics, Resource allocation, Q methodology

## Abstract

**Background:**

Many publicly-funded health systems apply cost-benefit frameworks in response to the moral dilemma of how best to allocate scarce healthcare resources. However, implementation of recommendations based on costs and benefit calculations and subsequent challenges have led to ‘special cases’ with certain types of health benefits considered more valuable than others. Recent debate and research has focused on the relative value of life extensions for people with terminal illnesses. This research investigates societal perspectives in relation to this issue, in the UK.

**Methods:**

Q methodology was used to elicit societal perspectives from a purposively selected sample of data-rich respondents. Participants ranked 49 statements of opinion (developed for this study), onto a grid, according to level of agreement. These ‘Q sorts’ were followed by brief interviews. Factor analysis was used to identify shared points of view (patterns of similarity between individuals’ Q sorts).

**Results:**

Analysis produced a three factor solution. These rich, shared accounts can be broadly summarised as: i) ‘A population perspective – value for money, no special cases’, ii) ‘Life is precious – valuing life-extension and patient choice’, iii) ‘Valuing wider benefits and opportunity cost - the quality of life and death’.

From the factor descriptions it is clear that the main philosophical positions that have long dominated debates on the just allocation of resources have a basis in public opinion.

**Conclusions:**

The existence of certain moral positions in the views of society does not ethically imply, and pragmatically cannot mean, that all are translated into policy. Our findings highlight normative tensions and the importance of critically engaging with these normative issues (in addition to the current focus on a procedural justice approach to health policy). Future research should focus on i) the extent to which these perspectives are supported in society, ii) how respondents' perspectives relate to specific resource allocation questions, and iii) the characteristics of respondents associated with each perspective.

## Background

All health systems face the same fundamental, moral dilemma: given that healthcare resources are scarce in relation to demand for them, how should they be allocated? This moral dilemma is also the defining social problem of the discipline of health economics. As advances in medicines, and in biotechnologies more generally, produce an increasingly ‘high-tech’, high cost range of available goods and services, and as pressure on public funding increases, debates about the fairness of healthcare provision have played out in the media as well as in academic and policy circles^a^. National health technology assessment agencies have proliferated, internationally, to evaluate the costs and benefits of medicines (and other technologies) and to make recommendations about the provision of healthcare^b^.

In the UK, as in many other countries, health technology assessment agencies have adopted a cost-utility framework for the economic evaluation of health technologies; typically operationalised as a cost per quality-adjusted life year (QALY) approach. The principles underlying that framework are related to opportunity cost and value for money. Technologies that produce more units of health per unit of cost are preferred to technologies that are less effective and less efficient. All else equal, patients with greater capacity to benefit will be prioritised over patients who will benefit less. This has distributional consequences and the issue of ‘special cases’ or, in other words, the question of when some health benefits are considered of greater value than others, has been debated for as long as health economists have been assessing costs and benefits. Many researchers have employed preference elicitation techniques to measure societal values with respect to different types of health gains arising to different groups of others [1,2]. Most recently, debate and research activities in this area have centred on the relative value of life-extending medicines for people with terminal illness (see for example the special issue of Health Economics Policy and Law [3]).

A handful of empirical studies have contributed to an evidence base around societal values on the topic, with a focus on whether there is a positive, end of life ‘premium’ or ‘QALY weight’ that would imply that health gains for people with terminal illnesses are associated with higher societal value than health gains benefiting other groups of patients^c^. The evidence, however, remains limited and equivocal. Shah *et al.* [4], Olsen [5] and Linley and Hughes [6] find no, or limited, support for an end of life premium. In contrast, Pinto-Prades *et al.* [7], Pennington *et al.* [8], Shah *et al.* [9] and Rowen *et al.* [10] find evidence suggestive of an end of life premium. These studies have taken quantitative approaches, including discrete choice survey methods [4,10], person or benefit trade-off questions [6,7] and willingness to pay (WTP) [7,8].

The nature of the empirical evidence highlights the need for further research investigating societal views around the relative value of end of life treatments for patients with a terminal illness. To date most studies have taken quantitative, choice-based or equivalence (matching) approaches to preference elicitation. In these studies, scenarios are constructed on the basis of a restricted number of attributes and present an artificial, controlled version of the issues at stake. Such reductionism is appropriate and necessary in studies that attempt to isolate the relative impact of a specific set of attributes on respondents’ choices. The range of different methods applied may partially explain the conflicting findings between studies but in-depth, qualitative exploration could provide richer insights into the reasons for disparities. Qualitative research may even suggest a wider set of relevant attributes than hitherto considered in quantitative studies. However, to date, qualitative studies tend to be used only as a precursor to (or extended pilot test of) surveys and there has not been an in-depth study.

In this study we use Q methodological techniques to investigate the nature of UK societal perspectives in relation to the relative value of life extensions for people with terminal illnesses.

### The UK context

According to the National Institute for Health and Care Excellence (NICE) decisions on the appropriate allocation of resources are based on scientific judgments, supported by legal considerations and, importantly for this research, social value judgments [11]. NICE social value judgments are grounded in the views of the general public, garnered mainly through the work of the Citizens Council^d^, and represented in a set of principles articulated in a document which was originally issued by NICE in 2005, with a second edition in 2008, and currently under revision again [12,13]. In their social value judgment document, existing debates on utilitarian and egalitarian approaches to distributive justice are mentioned in brief but NICE places emphasis largely on procedural justice, based on Daniels and Sabin’s [14] ‘accountability for reasonableness’. Despite the emphasis given to procedural justice or ‘process values’ [15], the cost per QALY calculation^e^ appears to remain central to NICE recommendations [16].

In 2009 NICE issued supplementary guidance for the appraisal of life-extending, end of life treatments [17] setting out criteria which permit such treatments to be recommended, even if they are not cost-effective according to the usual threshold applied.

### NICE Supplementary Guidance for End of Life Treatments

Treatments for terminal illnesses are non-curative by definition and so are likely to result in relatively small health gains when compared to other types of treatment. Such health gains often come at high cost and hence medicines of this type are less likely to meet NICE’s cost-effectiveness threshold. NICE technology appraisals rejecting a number of end stage cancer drugs, on the grounds of cost-effectiveness, were challenged and debates culminated in the Richards Report “Improving access to medicines for NHS patients” [18]. In response NICE issued their supplementary guidance for the appraisal of life-extending, end of life treatments [19]. This stipulates that such treatments may be recommended if all of the following criteria are met:

- “The treatment is indicated for patients with a short life expectancy, normally less than 24 months and;
- There is sufficient evidence to indicate that the treatment offers an extension to life, normally of at least an additional 3 months, compared to current NHS treatment, and;
- The treatment is licensed or otherwise indicated, for small patient populations” [17].

In a review of decisions following implementation of the supplementary guidance, Longson and Littlejohns [20] found that the NICE Appraisal Committee had recommended treatments at a cost per QALY which implied a QALY weight of 1.7 for life-extending, end of life treatments. On the basis of appraisal decisions between 2009 and 2011, Collins and Latimer [21] estimated that between 5,933 and 15,098 QALYs were displaced by the provision of end of life medicines (depending on application of a cost per QALY threshold of £30,000 or £20,000). The cost was estimated to be approximately £549 million a year, calculated using estimates of patient populations and incremental costs of treatment.

The Richards Report found a general view among stakeholders^f^ that “drugs to treat patients in the last months or years of life should be regarded as having a very high priority” ([18], p42). Of 29 NICE Citizen Council members, 10 thought it was acceptable to provide treatment above the threshold to extend life [22]. Additionally, a consultation conducted by NICE, prior to the implementation of the supplementary guidance, found some support for the guidance [23]. The nature of the support appears to relate in part to the failure of existing evaluation methods to capture fully the value of life-extensions for patients at the end of their life. Significantly, the consultation also revealed concerns regarding the lack of evidence to support the guidance “particularly on societal preferences with respect to end-of-life” ([24], p5). Nevertheless senior representatives of NICE maintained a view that, “the public, generally, places special value on treatments that prolong life – even for a few months – at the end of life” ([25], p348]). In 2014 the Scottish Medicines Consortium also introduced changes to their procedures for end of life and very Rare Conditions (orphan and ultra-orphan medicines) [26].

Despite a growing evidence base, in order for NICE, SMC and others to reflect the views of the public, more information is needed about the nature of societal values on the subject of life-extension at the end of life. This paper reports findings from a study^g^ designed to investigate UK societal perspectives on the relative value of end of life technologies.

## Methods

### Q methodology

Q methodology [27,28] combines qualitative and quantitative methods to study ‘subjectivity’ (subjective opinions, values or beliefs). These methods enable the identification and description of *shared views* around a given topic, in this case the relative value of life-extending treatments for people with a terminal illness. Q studies are comprised of two main features, a card sort used to generate data, and factor analysis to identify patterns of similarity between sorts. The first stage in Q studies is to derive a set of statements which is representative of the conversational *possibilities* (statements of opinion) in relation to the topic [29]. Respondents are then guided through a card sorting process known as a ‘Q sort’ in which they rank order the statements onto a grid according to a condition of instruction - for example, to arrange the statements from ‘most agree’ to ‘most disagree’. In the second stage, respondents’ Q sorts are subjected to by-person factor analysis, which identifies underlying dimensions in the data (factors) connecting similar Q sorts. By averaging similar Q sorts, factors are represented as a composite Q sort, which is a distinctive ranking of the original set of statements for each factor. This composite Q sort forms the basis of interpretation, the objective of which is to produce rich descriptions of each shared perspective (factor) on the topic of study.

### The statement set

There are two main concerns in the design of a statement set: coverage and balance [27]. Statements should be expressions of opinion or belief (not fact) and be relevant to the topic of study. The final set should avoid repetition and overlap and is intended to be *representative* of the population of opinions that exist in relation to the area of study. Methods used to generate a statement set aim to ensure that no relevant opinions are omitted. In the first instance all that is required for a statement to be included is that it states a subjective view on the topic in question.

In this study there were four sources of statements: the popular media, a public consultation conducted by NICE prior to instituting their supplementary guidance on appraising life-extending end of life treatments, qualitative interviews with key informants and focus groups with members of the general public.

We identified a number of newspaper articles reporting decisions of NICE committees regarding the approval or rejection of new ‘end of life’ drugs or treatments. From there we adopted a snowballing search strategy making use of hyperlinks to similar newspaper and website articles. We also searched articles from specific broadsheet and tabloid newspapers (selected to enhance the possibility of including a wide range of views) published around the dates of NICE decisions on end of life drugs. In total we extracted statements directly from the main text of 45 newspaper articles and from readers’ comments which followed. The NICE public consultation in 2008 returned over 800 statements from 300 respondents representing different types of stakeholders [30,31]. Finally, we conducted 16 interviews and three focus groups involving 20 participants. We began by interviewing a convenience sample of participants with personal experience of ‘end of life’ (for example bereaved family members, individuals from a cancer charity, a patient group) and then targeted individuals with relevant professional expertise; this latter group included health and medical professionals, an academic health economist, the pharmaceutical industry and a religious leader. Interviews followed a topic guide using open-ended questions to elicit statements of belief and opinion from respondents. A survey company recruited members of the general public, based on age and sex, to take part in focus groups. These were specifically aimed at eliciting views felt to be underrepresented in the sample of statements already collected, such as around reasons for valuing end of life treatments more than other health gains.

Examination of all sources returned more than 200 statements of belief or value related to the research question. Statements were then categorised using topic codes. Duplicate statements and those statements judged to be of secondary relevance to the research question were deleted, a process undertaken by two researchers (NMc and RB). For example, statements related to the reallocation of resources from other areas of public spending or the role of pharmaceutical companies in drug pricing were excluded because, although interesting, they were not directly related to the research question. Similar statements were merged and a pilot Q set of 65 statements was reduced further, through discussion with the wider research team and piloting with a convenience sample of 15 respondents, to a Q set of 49 statements (see column 2 Table 1).

**Table 1:** **Q set and factor scores**

**#**	**Statement**	**Factors**		
		**F1**	**F2**	**F3**
1	It is not worthwhile devoting more and more NHS money to someone who is going to die soon anyway	0*	−5*	2*
2	We should support an individual patient's choice for treatments that give short life extensions	−3*	3*	−1*
3	Treatments should be directed towards people who have a greater chance of survival	3*	−1*	0*
*4*	*If a special case can be made for expensive treatments for people who are terminally ill, an equal case could be made for drugs which slow dementia, help preserve eye sight, or reduce disability in children*	*3*	*1*	*2*
5	At the end of their life, patients should be cared for at home with a better quality of life rather than have aggressive and expensive treatments that will only extend life for a short period of time	4*	0*	1*
6	If somebody wants to keep fighting until the last possible moment, they should be allowed to do so, regardless of cost	−4	1*	−5
7	It's important to respect the wishes of patients who feel they should take every opportunity to extend their life because of their cultural or religious beliefs	−2	1*	−3
8	Patients should have the right to refuse life-extending treatments if they choose	4	5	5*
9	It is important to offer life-extending treatments to patients who have only recently found out that they are going to die soon	−2*	0*	−1*
*10*	*People should accept that if it's your time to die, it's your time*	*−1*	*−1*	*−1*
11	It is important to give a dying person and their family time to prepare for their death, put their affairs in order, make peace and say goodbyes	1*	4	3
12	I would value life extending treatments only if patients get a meaningful length of time - not just a few weeks	2*	−1	−1
13	I would place more value on end-of-life treatments than many medical treatments for non-terminal conditions	−4*	−2	−2
14	People who will die prematurely deserve to take priority over those who have non-life-threatening illnesses, even if it's not such a 'good' use of NHS money	−5*	−1*	−3*
15	Expensive drugs for people who are terminally ill and won’t benefit very much are not a good use of NHS money	2	−3*	3
16	It is human nature to want to preserve life and go on living for as long as we can - it is one of our most basic instincts	−1*	3*	1*
17	If a life-extending treatment for terminally ill patients is expensive, but the only treatment available, it should still be provided	−3	3*	−3
18	It may not sound like much, but a few extra weeks or months might mean an awful lot to a family affected by a terminal illness	0*	4	3
19	Life should only be extended if the patient’s quality of life during that time will be good	3	1*	3
20	We all have the right to life	1*	4*	−1*
21	We all pay for the NHS so we should have a right to life-extending treatment when we need it	−4	2*	−3
22	Real help and compassion should be about providing a death with dignity instead of more drugs to get a few more weeks or months out of a very sick body	4	0*	4
23	A year of life is of equal value for everyone	0	0	−4*
24	You can't put a price on life	0*	3*	−3*
25	We should spend proportionately more on patients when we feel those patients have not had their fair innings - in terms of the length of their life or the quality of that life	−3*	−1*	2*
26	It is wrong to raise hopes and expectations by making a special case for treatments that will only extend life by a short time	3*	−2*	0*
27	To extend life in a way that is beneficial to the patient is morally the right thing to do	−1	3*	0
28	If the means of helping someone live longer exists, it is morally wrong to deny them the treatment	−3	1*	−4
29	Not giving access to life-extending medicine to a person with a terminal illness is the same as killing them	−5	−2*	−5
30	The NHS budget is like a cake. Lots of small portions will do no good but if they are too big only a few people benefit. The simple answer is not to pay for very expensive drugs which only benefit a few	1*	−3	−2
31	Treatments that are very costly in relation to their health benefits should be withheld	1*	−5*	3*
32	People with a diagnosis of a terminal illness know that they will die early. Other people may not know in advance that they will die (e.g. patients with heart problems). It is unfair to give priority to those whose time of death is more likely to be known	0	−3*	1
33	End-of-life drugs are not a cure, they are life-prolonging. There is no point in delaying the inevitable for a short time	1*	−4*	−2*
34	Patients at the end of life will grasp any slightest hope but that is not a good reason for the NHS to provide costly treatments that may extend life by a short time	2*	−3*	4*
35	NHS provision of life-extending treatments should be decided on the basis of their cost and health benefits	5	−2*	4
36	Treatments that provide a short life extension are not worth it - they are only prolonging the pain for the patient’s family/friends	−1	−4*	−2
37	All human life is precious	1	5*	0
38	The health system should be about getting the greatest benefit overall for the population	5*	0	2
39	Extending life for people with terminal illness is only postponing death	0	−3*	0
40	Life is sacred and if it is possible to preserve life, every effort should be made to do so	−3	1*	−4
41	I wouldn’t want my life to be extended just for the sake of it - just keeping breathing is not life	3*	0*	5*
42	Treating terminally ill patients as more 'worthy' of NHS money undervalues the health of other NHS patients	2*	−2*	0*
43	Everyone has a right to basic healthcare but there has to be limits and expensive, end-of-life, drugs are not basic care	2*	−1*	1*
44	An end-of-life treatment which extends life-expectancy by 3 months now, is more valuable than a treatment for a long-term illness which extends life-expectancy by 3 months in ten years time	−2*	0*	2*
45	New breakthroughs are made every day - where there’s life there’s hope	−1	2*	−1
46	A few weeks of extra time is more valuable when patients only have a short time left	−1*	2	2
47	It's important to provide life-extending treatments to give a dying person time to reach a significant milestone, like a family event or a personal achievement	−2*	2	1
48	I think life-extending treatments for people who are terminally ill are of less value as people get older	−2*	−4*	1*
49	Treating people at the end of life is not going to result in big health gains but the health system should be about looking after those patients in greatest need	0	2	0

*denotes those statements that distinguish each factor from the other two factors (p < 0.01).

Italicized statements are consensus statements that do not distinguish between any two pairs of factors (non-significant at p > 0.01).

### Data collection

Q methodology sampling techniques are similar to those of qualitative studies and participants are selected purposively to identify data-rich respondents. Respondent sampling does not aim to achieve representation of a population, but rather to include those people who have rich, strong and different views on a subject. Sampling ceases when a ‘stable’ set of viewpoints are identified and additional participants serve only to confirm existing factors; typically occurring with 40–60 respondents [27]. In this study, individuals were selected who had different types of experiences or expertise in ‘end of life’ whether in a professional or personal capacity, who we would expect might take different positions on the subject. Thus we targeted individuals and groups connected, but not limited, to: academia, the pharmaceutical industry, charities and patient groups, religious groups, clinicians and people with experience of terminal illnesses (see Table 2 for a more detailed account of participants’ expertise and experience).

**Table 2:** **Respondents’ expertise/ experience and factor association**

**Q sort**	**Formal expertise/association**	**Experiential/ belief/personal**	**F1**	**F2**	**F3**
EX044	Health services research/health policy	Patient family	0.81*	0.10	0.17
EX045	NHS support worker	Patient/Carer of cancer patient	0.79*	−0.19	0.03
EX037	General Practitioner		0.79*	−0.00	0.19
EX005	Outcomes researcher/pharmaceutical industry		0.75*	0.04	**0.46**
EX023	Health care law/bioethics/NHS		0.75*	0.02	**0.42**
METAGP2			0.74*	0.14	**0.39**
EX033	Health economist/health policy/government		0.70*	−0.20	**0.42**
EX054	Politician	Patient family	0.70*	0.29	0.13
EX057	Journalist		0.69*	−0.31	0.20
EX017	Ethics and law/academic		0.69*	0.23	**0.40**
EX031		Patient family	0.69*	−0.15	0.26
EX040	Journalist	Patient family	0.69*	−0.27	**0.52**
EX003	Health economist/academic		0.67*	−0.03	0.15
EX015	Health economist/academic		0.64*	0.21	**0.55**
EX034	Health policy/charity		0.62*	**0.44**	0.36
EX058	Pharmacy/NHS		0.62*	0.11	0.27
EX056	Pharmacist/public health/NHS		0.52*	0.13	**0.47**
EX052	Oncologist		0.48*	0.23	0.36
EX036	Anglican Clergyman		0.42*	0.23	0.28
METAGP1			−0.14	0.92*	−0.19
EX030		Cancer survivor	−0.30	0.85*	−0.25
EX050	Nurse (cancer/palliative care)		0.03	0.76*	0.20
EX053	Health policy	Patient family	−0.18	0.72*	−0.00
EX038	Journalist	Patient	−0.27	0.67*	0.11
EX049	Sikh/charity		−0.06	0.65*	−0.12
EX029	Health policy/NHS manager	Patient	0.08	0.65*	0.14
EX010	Medical marketing		−0.20	0.62*	0.35
EX009		Experience of bereavement	0.22	0.61*	0.14
EX032	Public health/health policy/NHS		0.25	0.60*	0.25
EX041	Chaplain/hospice		0.21	0.59*	0.24
EX042	Health policy/hospice		0.15	0.58*	0.19
EX059	Muslim		0.03	0.54*	−0.17
EX022	Cancer charity/cancer nurse		0.32	0.46*	0.32
EX018	Cancer nurse		0.15	0.45*	**0.39**
EX024	Economist/industry		−0.09	0.25	0.77*
EX014	Health economist/academic		**0.37**	0.30	0.68*
EX011	Health economist/academic		0.25	0.26	0.66*
EX026	Health economist/academic		**0.38**	−0.09	0.65*
EX055	Oncologist		0.03	0.27	0.65*
EX025	Health economist/pharmaceutical industry		**0.44**	−0.05	0.63*
EX001	Ethics/academic		0.27	0.10	0.63*
EX007	Health economist/NHS		0.29	0.17	0.58*
EX028	Health economist/academic		0.33	0.14	0.58*
EX021	Health economist/academic		0.30	0.10	0.54*
EX047	Palliative medicine/charity		**0.43**	0.16	0.53*
EX016	Health economist/academic		**0.43**	0.12	0.52*
EX043	Nurse (cancer/palliative care)/charity		0.33	0.14	0.52*
EX002		Experience of bereavement/dignitas member	**0.50**	−0.03	0.51*
EX004	Medical ethics/academic		0.23	0.21	0.43*
EX020	Public health/health policy/NHS		0.22	0.16	0.38*
EX006	Medical advisor/pharmaceutical industry		0.33	0.34	**0.45**
EX008	Health technology assessment/pharmaceutical industry		**0.48**	0.28	**0.51**
EX012	Cancer research/academic		**0.57**	0.17	**0.57**
EX013	Health economist/NHS		**0.59**	−0.27	**0.58**
EX019	Health economist/academic		**0.52**	−0.34	**0.58**
EX027	Bioethics/academic		**0.49**	0.13	**0.48**
EX046	Medical consultant/palliative medicine		**0.50**	0.32	**0.58**
EX035	Health policy/charity		0.34	**0.38**	**0.50**
EX048	Nurse/health policy	Patient	**0.38**	0.29	**0.46**
EX051	Cancer nurse		**0.44**	**0.41**	**0.44**
EX060	Muslim		**0.38**	0.24	**−0.40**
**% exp. Var.**			22	14	19

The significance level for factor loadings is taken as 2.58 (SE). SE represents standard error that is defined as 1/√N where N is the number of statements in the Q set. In this case then, 2.58 (SE) = 2.58 (1/√49) = 0.37. Significant loadings are shown in bold type.

The automatic flagging procedure in PQ method software was used to identify defining sorts (*) according to the following rule: Flag loading *a*: *if* (1) *a* ^2^ > h^2^/2 (factor ‘explains’ more than half of the common variance) and (2) *a* > 1.96/√(N items) (loading ‘significant at p <; .05’).

As noted previously, this Q study was the first phase of a larger project, the second phase of which is a survey designed on the basis of findings from phase 1. For that reason, we also conducted the Q sort in a general population sample, selecting 250 members of the general public using ‘non-Q methodological’ sampling methods. This respondent group was quota-sampled from 10 regions, with a representative urban and rural split, across the four countries of the UK. Quotas were set for age, gender and employment status and the sample was monitored for representation of religion, ethnicity and self-reported health.

**Figure 1:**
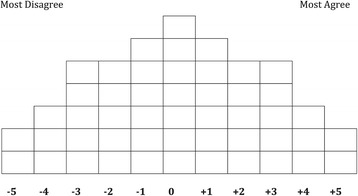
**Q Grid.**

Each respondent completed a Q sort, administered by a member of the research team in the ‘purposive’ sample and a trained interviewer^h^ in the ‘General Public’ sample. An introduction outlined the focus of the study (see Appendix 1) then respondents were issued with a pack of 49 shuffled statement cards (Table 1) and asked to consider each statement in turn and to assign it to one of three piles: agree, disagree, or neutral. Respondents were then guided through the rank ordering of the statements using the Q grid (see Figure 1). Each space in the Q grid indicates a ranking ranging from −5 (most disagree) to +5 (most agree); all statements within a column are equally ranked. Respondents selected two statements to place in the two ‘most agree’ (+5) spaces on the grid, then three statements for position +4 and then five statements for the +3 position. Switching to the ‘disagree’ side of the Q grid respondents followed the same process, from the left hand side, until statements had been placed in the −5, −4 and −3 columns. Respondents completed the remaining grid, column by column, until all 49 statements had been positioned.

Following the completion of the Q sort there was a short, audio-recorded interview during which respondents were asked to sum up their views on the issues raised and to comment on the statements placed in the extremes of the Q grid, in positions −5 and +5. Interviews were transcribed verbatim and transcripts stored in QSR NVivo 10 [32].

### Analysis

The aim of Q factor analysis is to identify underlying similarities between Q sorts that represent shared points of view. Q sort data were entered into a dedicated software package, PQMethod [33], and Centroid factor analysis was followed by Varimax rotation. Factor analysis will usually produce several statistically possible factor solutions. We selected the preferred factor structure (number of factors) based on the examination of statistical output and the coherence of accounts from the emergent factors [27].

Interpretation of factors involves the holistic interpretation and description of the perspectives represented by the composite Q sort of each factor, with reference to the relative position of statements within and between factors. Particular attention is given to salient statements, which are those in the outer columns of each side of the Q grid i.e. -5, −4, +4 or +5, and distinguishing statements, which are those with a significantly (p < 0.01) different position in the composite Q sort of a factor as compared to the other factors (see Table 1). Additionally, interpretation makes use of responses to the post Q sort qualitative interviews of respondents with a high factor loading on only one factor. These data often help to make connections, or offer explanations for why a statement is important or why the placement of two statements might be related.

### Research ethics

Ethical approval for this study was obtained from the School of Health and Life Sciences Ethics Committee, Glasgow Caledonian University (reference B11/04) and the work was undertaken in line with the principles of the Declaration of Helsinki [34]. Individuals identified as possible participants for interviews, focus groups or Q sorts were provided with an information document describing: the purpose and nature of the research; details about confidentiality and storage of data; ethical approval and funding of the project. All participants were given the opportunity to ask further questions or to seek clarification about any aspect of the study before deciding whether to take part. Participants were assured that they would not be identified in any project publications or other outputs, informed they were free to withdraw from the study at any time and that there were no right or wrong answers to the questions posed. Written informed consent was provided by all interview and Q sort participants prior to data collection. Verbal informed consent was provided by focus group participants.

## Results

59 Q sorts from the ‘purposive’ sample were analysed (see Table 3 for a summary of respondents’ characteristics). A range of factor solutions were examined and a three factor solution yielded interpretable accounts, which were consistent with the qualitative data. From the ‘General Public’ sample, factor analysis resulted in two factors which were highly correlated with the first two factors from the purposively sampled respondents; with correlations of 0.74 and 0.92 respectively. No additional viewpoints were identified in the general population sample and so we focus on the more detailed results from the purposive sample. The two viewpoints from the general population enter our analysis as composite Q sorts – a form of ‘meta-respondent’ (shown in Table 2 as METAGP1 and METAGP2) allowing the positioning of the three factors together with those from the public. Thus 61 Q sorts formed our final sample with the three factor solution remaining stable.

**Table 3:** Summary characteristics of full sample respondents (n = 59*) and respondents defining the factors

		**Full Sample**		**Defining Sorts**		
		**N**	**%**	F1 (n = 19**)	F2 (n = 15**)	**F3 (n = 16)**
**Age**	18 - 30	4	7%	1	1	1
	31 - 50	31	53%	8	6	10
	51 - 64	17	29%	8	5	2
	65+	5	9%	1	2	2
	Not stated	2	3%	0	0	1
**Gender**	Female	35	59%	11	10	7
	Male	24	41%	7	4	9
**Expertise**	NHS	22	36%	8	6	5
	Academic	14	24%	3	0	8
	Pharmaceutical Industry	6	10%	1	1	2
	Religion	5	8%	1	3	0
	Patient Groups (e.g. Cancer charities)	3	5%	1	1	0
	Journalist	3	5%	2	1	0
	Politician	1	2%	1	0	0
	Personal experience of terminal illness and/or bereavement	5	8%	1	2	1
Experience of Terminal Illness (all respondents)***	Cancer	30		10	10	5
	Other terminal illness	23		8	8	4
	No	16		4	1	8
	Unstated	1		0	0	0

Note *characteristics of the general public sample defining the two ‘meta-respondents’ 60 and 61 are available from the authors on request.

**This total includes a ‘meta-respondent’ for F1 and F2 respectively. Individual characteristics for ‘meta-respondents’ are not included in this table.

***Categories within ‘Experience of Terminal Illness’ are not mutually exclusive so responses will not sum to 59.

The factors represent three different perspectives, and all 61 Q sorts are, positively or negatively, to some greater or lesser degree, associated with all three accounts. Factors 1 to 3 were defined by 19, 15 and 16 Q sorts, respectively (see Tables 2 and 3 for more details). An explanation of the rule used to indicate a defining sort follows Table 2 (for example, respondent EX044 in Table 2, helps to define factor 1). Others do not define a factor and/or agree in part with more than one account and are known as ‘mixed loaders’ (for example respondent EX006).

Table 1 shows the composite Q sorts for the three factors. The correlations between composite Q sorts were −0.05 between factors 1 and 2, 0.68 between factors 1 and 3 and 0.09 between factors 2 and 3, indicating that factors 1 and 3 are similar (the similarities and differences between these two factors are drawn out in the subsequent factor descriptions). There were two consensus (non-distinguishing) statements across the three factors, #4 and #10.

In the following subsections each of the three factors is described with reference to the positioning of statements within each factor, and quotes from the post Q sort interviews are used to enhance both the interpretation and the description of these accounts. Notation is used in the descriptions below as follows: # indicates statement number, and is followed by the position of that statement on the grid for that factor. For example, (#38, +5) indicates that statement number 38 appeared in position +5 for the factor in question.

### Factor 1: A population perspective – value for money, no special cases

Factor 1 describes a system-level perspective in which provision of treatments and services within the National Health Service (NHS) should aim to maximise health benefits overall for the population, from the budget available, in order to best serve the interests of society (#38, +5). NHS resources should be allocated on the basis of the costs of treatment in relation to the health benefits that arise from treatment (#35, +5). Because the health system has limited resources with multiple potential uses, value for money is important (#15, +2; #14, −5). Hence treatments that yield the greatest health improvements with respect to cost should be prioritised. This overriding principle implies that, aside from their capacity to benefit, all patient groups are considered equally deserving of treatment, no matter what their age (#48, −2), type of condition (#13, −4; #14, −5, #4, +3) or ‘level of need’ (#49, 0).

*“people often say . . . . . they think it’s scandalous that something as unimportant as money should stand between somebody in life and death, and you can understand emotionally why that sounds right until they stop and think about, well money isn’t just money it’s somebody else’s opportunity for easement of pain or suffering or prolongation of perhaps a better quality of life”* (EX040).

The aim of maximising population health from the available budget means judging provision on the basis of expected health gains from treatment. People with terminal illnesses have, by definition, limited potential for health gains, but due consideration should be given to patients who do not have a terminal illness as well as those that do (#42, +2). Terminal illness should not be treated as a special case (#4, +3; #14, −5; #13, −4; #44, −2) and doing so would imply that health gains for people with terminal illness are valued more highly, devaluing the health of all other NHS patients (#42, +2).

*“These people do not deserve to take priority because everybody’s got the same right to be treated…”* (EX037)*.*

Life-extending treatments, which only provide a short life extension at high cost, should not be provided (#2, −3; #15, +2). Instead resources should be directed towards other patients with a greater chance of benefit (#6, −4; #3, +3). Prioritising life extending treatments could also raise false hopes among patients who may have high expectations of treatments that may not deliver on meaningful health benefits (#26, +3; #34, +2).

*"typically people have a perception they’ll get a lot more for using expensive drugs at the end of life than actually the evidence suggests that they will. And many people who talk about life extending treatment to the end of life, possibly actually think that they’re going to have their life saved and cured......actually it’s pretty unclear whether it will make a difference"* (EX023).

Although an individual may have paid into the system through their tax contributions, the NHS is a public resource designed to get the best overall health for the population and people do not have a right to claim life extending treatment particularly as these treatments are not ‘basic care’ (#6, −4; #21; −4; #43, +2). There is no inherent moral requirement, in this account, to provide treatments (#28, −3) on the basis that patients have not had their ‘fair innings’ in life (#25, −3); or because they have recently learned they have a short life expectancy due to illness (#9, −2); or having no other treatments available (#17, −3). Since the emphasis is on health benefits, it also follows that life-extending treatments should not be provided on the basis of non-health benefits, whether this be for religious or cultural reasons (#7, −2), to enable an individual to reach a significant milestone (#47, −2), or even because life is sacred (#40, −3).

Given that this account is focused on providing the most cost effective treatments, other (less effective, more costly) treatments would not be provided and hence this population approach undermines what some patients would regard as their individual right to access specific treatment options - unless those treatment choices would also maximise population health (#6, −4; #2, −3; #21, −4). Patients do not have a choice of all possible treatments, but across all factors there is agreement that they do have the right to refuse treatment if they choose (#8, +4). Life extending treatments are only of value if they are likely to have significant health benefits to the individual such as enhanced quality of life (#19, +3) and/or a meaningful life extension (#12, +2; #26, +3). An individual’s wishes to have a more comfortable and dignified end of life at home with a better quality of life is more important than aggressive and expensive treatments with little benefit (#22, +4; #5, +4).

### Factor 2: Life is precious – valuing life-extension and patient choice

This account is based on a belief that human life is precious, the desire to preserve life is inherent in human nature and the impulse to go on living as long as we can is a basic instinct (#37, +5; #16, +3). As such, everyone should have a right to life (#20, +4) and end of life treatments that result in a short life extension (and not a cure) are worth providing (#1, −5; #33, −4; #36, −4; #49, +2) even as people get older (#48, −4). Short life extensions for people who are terminally ill are viewed as preserving (#16, +3) and prolonging (#33, −4) life rather than postponing death (#39, −3).

From this perspective, cost effectiveness should not be the principal basis of coverage decisions (#31, −5; #24, +3) and just because treatments are expensive in relation to their benefits does not, for factor 2, mean they should not be provided (#1, −5; #34, −3; #15, −3; #35, −2).

*“I think it is wrong to withhold a life prolonging medication from someone on the basis of cost or age”* (EX050).

*“…we cannot completely ignore cost but it should not be our fundamental decision maker”* (EX050).

*“extending life for people with terminal illnesses is only postponing death? Well I would say that my feelings again are the reverse of that, that we're enhancing what life is left*” (EX029).

A key issue for factor 2 is individual patients’ choice. Patient choice should inform resource allocation decisions (#8, +5; #2, +3). This is an account grounded in rights-based arguments, which places the patient at the centre (#27, +3; #20, +4; #21, +2; #16, +3). Life-extending treatment should be provided if a patient wants it as everyone has contributed to the funding of the NHS (#21, +2). Crucially, this should apply not only to life-extending treatments for terminal illness, but also to other NHS patients with non-terminal conditions (#13, −2).

*“I think that if a system such as the NHS is to be truly compassionate, the patient choice and family choice has to be one of the premier things that we consider”* (EX009).

Short extensions to life may be valued for the non-health benefits to terminally ill patients or their families - helping them to prepare for life without their loved one, put their affairs in order, make peace, or say goodbye (#11, +4; #36, −4; #18, +4). Life extension, if it is beneficial to the patient, is morally the right thing to do according to this account (#27, +3).

*“if (someone wants) to achieve completion, resolution and closure around certain issues, I believe that they have a moral case for life-prolonging drugs”* (EX038).

Factor 2 preserves hope of cure if life can be extended for even a short period of time, here ‘where there is life there is hope’ and the potential for new breakthroughs (#45, +2; #26, −2).

### Factor 3: Valuing wider benefits and opportunity cost – the quality of life and death

Factor 3 is, like factor 1, an account based on concerns about achieving value for money with respect to NHS resources (#35, +4; #15, +3). Assessing the benefits of treatments in relation to costs will result in denial of treatments, and withholding cost-ineffective treatments is acceptable and consistent within this account (#31, +3). Individuals do not have a guaranteed right to be provided with any treatment that is available (#6, −5; #21, −3) because the effective use of limited NHS resources must be considered (# 14, −3; #35, +4; #31, +3; #34, +4).

“*We should be aware of what it is costing to keep us alive and what the cost to other people might be*” (EX002).

*“it’s important that NHS resources are spent in a way that is good value for money*” (EX014).

Unlike factor 1, this account also incorporates the value that patients and their families attach to life extension at the end of life. Thus, treatments which extend life by a short time, at high cost, could be (potentially) good value for money by accounting for the value placed on that time. Such value arises from non-health benefits such as preparing for death, making peace, spending time with family and saying goodbye (#11, +3; #18, +3;). Time itself could be more valuable when only a short life remains (#46, +2; #44, +2), but treatments for patients who are terminally ill are not regarded as more valuable across the board (#13; −2; #14, −3) because quality of life and quality of death are important (#22, +4; #19, +3). For factor 3, not all treatments at the end of life are more valuable *per se*, but some treatments, even those associated with small health gains, may yield other benefits to patients and families which are of great value at the end of life. It follows that there may be benefits that are not currently captured in cost effectiveness analyses.

Respondents who are associated with factor 3 place great importance on *not* extending life for the sake of it and patients’ right to refuse treatment (#41, +5; #8, +5), a concern which is consistent with an emphasis on the importance of quality of life and dignity in dying (#19, +3; #22, +4).

*“we don't run society just for the aim of maximising the person-years that can be lived and .. just because something is technically possible, it doesn't mean that that should be done”* (EX014).

*“we should think about how people die and not just try and pretend it doesn’t happen or it’s not going to happen”* (EX011).

*“death is now a huge taboo and a huge fear and so the end of life is looked on with horror and it should not be”* (EX002).

This account holds that there are cases where more value should be placed on time at the end of life than for other patients, at other times (#23, −4; #44, +2; #46, +2). However, the value of life extensions for people with a terminal illness is not unconditional and provision should not be based on an individual’s cultural or religious beliefs (#7, −3) or desperate hopes (#34, +4), there must be a worthwhile benefit (#40, −4; #15, +3), as opportunity cost needs to be considered.

*“opportunity cost matters, treating terminally ill patients as more worthy of NHS money, undervalues the health of other patients? Yes it does because you have to say to somebody else, ‘we think they're more valuable and therefore you're less valuable’”* (EX026).

## Discussion

The aim of this study was to identify and describe the societal perspectives that exist around the relative value of life-extending treatments for terminally ill patients. The empirical literature had previously not explored qualitatively and in-depth the views that exist, instead studies have been quantitative, choice based or equivalence approaches to preference elicitation. Their equivocal findings highlighted the need for more extensive qualitative exploration to help elucidate possible reasons for disparities in quantitative findings. The three shared perspectives found in this study highlight the plurality of views and the importance, for both researchers and policymakers, of a greater understanding of the issues surrounding this topic.

### Discussion of the factors

Factor 1 closely resembles a utilitarian perspective. The central principle is to achieve the greatest health gains for the greatest number through the efficient allocation of limited resources. People who are strongly associated with this point of view would be unlikely to support a policy which gave extra weight to relatively small health gains that result from life-extending treatments at the end of life and hence the supplementary guidance for end of life treatments [17] would be unlikely to meet with the approval of individuals associated with factor 1.

Factor 2 is wholly different from factor 1. The utilitarian tendencies of factor 1 mean that restricting the availability of cost-ineffective treatments is acceptable, whereas factor 2 respondents reject the denial of life extending treatments to patients with terminal illnesses. In this account patients’ rights are central and life is regarded as precious and priceless so even high cost treatments that deliver limited benefits should not be withheld from patients. This account is consistent in that treatments should be provided if patients and their families feel they will be beneficial and that patients should still be able to refuse life-extending treatments if they choose. Yet it is unclear whether people who align with factor 2 would support the existence of a special policy for end of life medicines. This is not because they believe these treatments should not be provided but rather they appear to disagree with cost effectiveness analysis altogether as a means of guiding provision and oppose restriction of access to any treatments for any patients who want them, regardless of whether they are terminally ill or not.

Factor 3 appears to be a more nuanced and balanced perspective as it supports value for money, but that value is broader than health gains and partly defined by individual (over societal) preferences. Life-extending treatments may be very valuable (and hence should be provided) but, crucially, value is contingent on quality of life and life should not be extended for the sake of it. Efficiency is an important aspect of this account, and patients do not have a right to any and all treatments, some of which will be withheld on the grounds of cost effectiveness. Given the emphasis on quality of life, it is unlikely that respondents who define factor 3 would support a policy such as the NICE supplementary guidance which prioritises life-extending treatments and does not take into account quality of life. However they would likely support a policy that incorporated wider benefits of value to patients and families at the end of life. Whether that would be achieved by weighting QALYs is not clear from this analysis alone.

A qualitative sample, such as this, does not permit generalisations about the characteristics of respondents who are associated with each of the factors. However, interesting observations can be made about the *types* of respondent defining each factor as a potential means of enhancing our interpretations. For example, Table 3 shows that no academics help to define factor 2 in our sample, while factor 3 has the greatest number of academic respondents. Of the academic respondents strongly associated with factor 3, six of the eight respondents have expertise in health economics; two are ethicists. This does not imply that, in general, academics or health economists will express a view such as factor 3, but it can be brought to bear in our interpretation of the factors. In addition, fewer factor 3 respondents have had experience of a close relation or friend suffering a terminal illness.

### Ethical and policy implications

The ethical and policy implications of the accounts identified here relate, in part, to the status accorded to those accounts – and to whether they can be considered societal views. The factors identified, although drawn from a relatively small, purposive sample of respondents, are nevertheless views that are held within society. Indeed these views emerge from studying individuals who, we would argue, are best placed to articulate a clear and considered perspective on this topic given their experience or expertise. This observation might explain why an additional factor was found in the purposive as compared to the general public sample which, conversely, was quota-sampled to reflect certain characteristics of the UK population. An alternative or supplementary explanation for this finding is simply that quota sampling is not an efficient way to identify the nature of views in society, for which purpose qualitative sampling techniques are better suited. General population sampling methods might result in a number of people with strong and different views on the topic of interest, but it is equally likely not to locate those views, unless they are strongly associated with other, particular characteristics which happened to have entered the sampling frame (for example viewpoints that strongly associate with age or ethnicity or gender).

Despite all of this, we were admittedly concerned that we might have ‘missed something’ and so included the general public sample as an additional ‘check’. It was reassuring to note that there were fewer (not more) views discernible in the less purposeful sampling. However, the mixed approach we have taken in this study raises questions about the status of the factors derived from each sample and, in particular, whether the three factors we describe from a sample of 59 respondents can be described as ‘societal views’. In our view they can - since we identified those members of society with strong and different views and looked for shared perspectives amongst them. To what extent ‘society’ (i.e. a large, representative sample of the public) supports each of the views or how well-represented a community is by one or more viewpoints are separate (though related) questions.

It is interesting to note that the main philosophical positions that have long dominated debates on the just allocation of resources have a basis in public opinion. This is most apparent in the utilitarian focus of factor 1 and concern for the intrinsic value of life and patient rights prominent in factor 2. Although factor 3 exhibits a greater degree of moral pluralism, concerns with quality of life and appropriate cost/benefit distribution also represent ethical and economic issues that are of central importance within policy debates.

Beyond this basic concurrence with theories, the factors present a number of challenges that need to be addressed if they are to support commitments to utilise public opinion to inform policy and practice. Firstly, for people associated with factor 1, end of life health gains will never be regarded as inherently more valuable than other treatments yielding greater health gains and competing for funding. For factor 3 the picture is less extreme and, in the event that life extension yields real benefits (which may or may not be related to health) and good quality of life, the value placed on that time by patients and their families may mean that it is good value for money. Factor 2 gives a relatively low ranking to a number of statements pertinent to the importance of end of life treatment (#13, −2; #14 -1). It seems that within factor 2 the placing of these statements is determined by that factors deference to patient choice; the relative value of end of life treatment is determined by whether individual patients want to avail themselves of it – if they do, it should be provided regardless of cost. These emergent views, taken together with other recent empirical research, the limited support provided by NICE’s Citizens Council and the consultation on the supplementary guidance, suggest that, if societal perspectives are a key element in health policy decisions, further consideration should be afforded to the way resources are allocated to patients at the end of life. NICE is in the process of considering new appraisal methods (at the time of writing these are still under consultation but include measures of absolute and proportional QALY shortfall) which would suggest that patients’ QALY loss in terms of both length and quality of life could be taken into account [35]. It is not clear whether the end of life supplementary guidance will be supplanted by this. Nevertheless, to uphold their commitment to procedural justice it is necessary for NICE to provide a transparent account of *how* public values are incorporated into processes.

Secondly, although the factors proffer a reliable, snap shot of the diverse ethical positions that exist amongst the public, the perspectives do not present ready-made policy positions. The existence of certain moral positions does not ethically imply, and pragmatically cannot mean, all are translated into policy. This is apparent in the diverse nature of often mutually exclusive standpoints and as such ways must be found to weigh and choose between them. Hence, it is important to know how strongly these views are supported, and how they relate, for instance, to political or wider social value orientations.

In addition, despite its democratic appeal, determining policy by means of a simple majority vote could result in policies that infringe rights and freedoms in ways that would offend fundamental liberal sentiments [36]. A subsequent ramification is that it does not necessarily follow that because two of the three factors (and other empirical information) are against giving greater value to end of life treatments, that the position should be adopted. Rather, normative judgments must be made about which views are most appropriate in terms of ethical suitability and viability for public policy and services. Emergent factors can help to inform such analyses, but in order to advance critical engagement with the ethical convictions of citizens policy makers need to engage with, and not stand back from, the normative positions identified within the factors.

Engagement with, and critical examination of, public opinion is important from a public perspective to ensure society develops in ways broadly in keeping with its fundamental convictions and to engender citizens with the implications of different ethical positions, within end of life policy and beyond. The need for this type of work can be illustrated through a number of issues central to debates at the end of life and within the factors; namely, health maximisation and citizen choice. Regarding efforts to maximise health gain, factor 1 represents a position that aims to achieve the greatest overall benefits from the limited resources that are available. In places the factor appears to suggest that such an approach is impartial and even-handed (#4 + 3). Thus, as Sen notes, this type of utilitarian position has gained an “ill-deserved egalitarian reputation” ([37], p16]). A health system based on utilitarian principles has distributional consequences in that it will implicitly discriminate against individuals who will return fewer benefits from the investment of resources obtained. Such partiality is apparent in “utilitarianism ageism” that prioritises “the greater expected duration of health benefits in young people that derives from their greater life expectancy” ([38], p103); and can also be extended to others who can be thought of as receiving less benefit, including the disabled or impaired [37,39] and those with terminal illness. Difficulties emerge regarding how to make valid judgements about the value of life to different individuals (or sectors of the population) without unfairly discriminating, or using assumptions that may be flawed - such as the young will actually live longer (and so benefit more) than those who are elderly [40].

In respect of individual choice, we have seen that factor 2 gives priority to individual rights and decisions regarding end of life treatment. However the factor affords equal importance to the statements all life is precious (#37, +5) and that patients should be able to choose to end their lives by refusing treatment (#8, +5). Within the ethics literature these positions do not always align easily with each other. This is because for some people, an implication of all life being precious (#37, +5) is that we also have a basic instinct to preserve life (#16, +3). People who hold this position are likely to see individual decisions to refuse treatment that will end a person’s life as being ethically unacceptable. This is because to them choosing to end life vitiates against the idea that life is precious. For example, within a deontological position, with which factor 2 has much in common, there are moral limits on patient or citizen choice. It has been argued, for example, that given the value of human life the rationality of seeking to end such intrinsic goods may undermine fundamental human duties [41].

Further, there exist practical limitations to support individual choice, despite factor 2’s preference for it to be unconstrained. Namely, it is not feasible for states to meet all needs or wants due to constrained resources. It seems factor 2 respondents fail to acknowledge the reality of budget constraints and the opportunity cost of coverage decisions (whether explicitly stated or not) despite emphasis placed on the need for health systems to make difficult decisions about a fixed budget (see the opening and closing paragraphs of Appendix 1). However, this ‘refusal to ration’ could be based on the belief that the treatments would be affordable if other measures were put in place. For example, some interview respondents suggested reallocating resources from other areas of government spending (e.g. defence), cutting waste in the NHS, or greater regulation of pharmaceutical industry pricing.

In liberal democracies the aim of supporting individual freedoms through public resources makes it necessary to achieve a balance between the public system (prioritised by factor 1) and individual wants (privileged by factor 2); an issue which both factor 1 and 2 overlook in favour of ceding to their driving principle. Factor 3 however acknowledges this aim in its recognition of a need for a broader and more inclusive ‘evaluative space’. Yet, this point of view could also prove the most challenging to operationalise as it is not clear what wider benefits of value to patients and families at the end of life should be considered, how they should be assessed and by whom.

## Conclusion

While this study has identified and described societal perspectives on the relative value of end of life technologies, not previously explored in other empirical studies on this issue, our findings highlight normative tensions that require further attention. Such tensions reveal the importance of critically engaging with, rather than adopting a procedural approach to, normative issues to ensure that public policy seeks as far as possible to promote fundamental convictions of society. Q methodology is an approach that can be employed to identify the issues or tensions that need to be critically debated and deliberated.

Furthermore, for societal perspectives to be valuable for policy it is necessary to understand the extent to which these views are held and how they relate to specific questions of resource allocation, wider social value orientations and other characteristics. The development of methods to investigate these issues will be undertaken and then employed in a nationally representative survey of the UK general public, conducted in phase 2 of this study.

### Endnotes

^a^The increasing level of technology in medicine is not the cause of this problem of scarcity, but may energise the issue with the emergence of new and expensive pharmaceuticals, diagnostic technologies and devices.

^b^Examples of such organisations are the Canadian Agency for Drugs and Technologies in Health (CADTH); the National Evidence-based Healthcare Collaborating Agency (NECA) in Korea; the Health Intervention and Technology Assessment Program (HITAP) in Thailand; the National Health Care Institute (ZiNL) in the Netherlands; and, in the UK - which is the context for the empirical work reported in this paper - the National Institute for Health and Care Excellence (NICE), the Scottish Medicines Consortium (SMC) and the All Wales Medicines Strategy Group (AWMSG).

^c^Note that ‘end of ‘life’ is often applied quite loosely in this literature. Typically it refers to both imminent and premature death (referring to people with a terminal illness that shortens their life expectancy and not to the very old who reach the end of a ‘natural life span’). This is also the context in which we use the term ‘end of life’ in this research.

^d^The Citizens Council, established by NICE in 2002, is an assembly of 30 rotating members from a variety of backgrounds that debate and provide opinion on values-based challenges with a bearing on the allocation of health technologies.

^e^Quality Adjusted Life Years (QALYs), combine measured changes in the quality and quantity of life and are used to assess the health benefits of treatments. The findings of cost-utility analyses are typically presented in terms of the cost per QALY gained. NICE apply a cost-effectiveness threshold (range) of £20,000-£30,000 per QALY gained, above which technologies are usually not recommended for NHS funding unless a special case is made for their provision [25,42].

^f^This includes, but is not limited to, the public, patients, NHS managers, healthcare professionals and the pharmaceutical industry.

^g^This study was funded by the Medical Research Council Methodology Panel and represents the first stage of a two-part study. In this phase of work we applied established methods (Q methodology), in the second phase we explored different survey methods to investigate the prevalence and distribution of the societal perspectives identified and reported here.

^h^A survey company (IPSOS MORI) were commissioned to conduct the 250 Q sorts with the general public. Interviewers were trained by the research team to administer the Q sorts and also observed Q sorts with pilot respondents.

## Appendix 1 Introduction

The National Health Service only has so much money available. There are many things that the NHS could spend the money on like new drugs, treatments, doctors, nurses, beds and so on. Unfortunately not everything can be funded, no matter how large the budget.

You may have views about other government spending – but this study is concerned with how the health budget is spent, so we’d like you to focus on that.

These are difficult decisions. The cost of each treatment and how much good it does have to be considered, as do views about what else might count when deciding how to ‘cut the NHS cake’ - for example, whether to spend money treating those people who are the most severely ill; or favouring treatments that give patients a better quality of life; or else treatments that help them live longer.

It is important that the general public has a say as they pay the bills, benefit from the services provided and do not benefit if a service is not provided.

In this project, we are interested in what you think about funding treatments that help terminally ill patients to live longer.

These treatments will not cure their illness, but they will extend their life, usually by weeks or months. These treatments have to be paid for and if money is spent on these treatments it would not then be available to spend elsewhere in the NHS.
